# Phenotype to Treatable Traits-Based Management in Chronic Obstructive Pulmonary Disease

**DOI:** 10.7759/cureus.60423

**Published:** 2024-05-16

**Authors:** Ankit Kumar, Surya Kant, Vijeta Niranjan

**Affiliations:** 1 Respiratory Medicine, King George's Medical University, Lucknow, IND; 2 Pathology, T.S. Misra Medical College and Hospital, Lucknow, IND

**Keywords:** expectoration, pulmonary exacerbation, dyspnea, bronchiectasis, fixed airflow limitation, emphysema, chronic bronchitis, treatable trait, copd (chronic obstructive pulmonary disease)

## Abstract

Chronic obstructive pulmonary disease (COPD), a heterogeneous respiratory disease driven by various genetic and environmental factors, presents significant challenges in diagnosis and management. Traditional approaches focused on phenotypic classification, but recent paradigms emphasize identifying and addressing treatable traits to personalize treatment strategies. Treatable traits facilitate personalized interventions, optimizing symptom control, and reducing exacerbation risk. Dyspnea and exacerbations, recognized as key traits, guide treatment decisions and follow-up management. Various interventions, including bronchodilators, corticosteroids, and lifestyle modifications, target specific traits like airway inflammation, mucus overproduction, and emphysema. Strategies for assessing and addressing treatable traits during initial encounters and follow-up visits enhance disease monitoring and treatment efficacy. Comprehensive trait assessment demands resources and specialized monitoring, posing barriers to widespread implementation. The lack of standardized protocols and evolving evidence further complicates decision-making and clinical practice. Despite these challenges, the shift toward treatable traits-based management signifies a pivotal advancement in COPD care, emphasizing holistic approaches tailored to individual patient needs. Recognizing and addressing treatable traits offers personalized interventions, enhancing symptom control and disease management. Embracing treatable traits-based approaches holds promise for improving clinical outcomes and enhancing the quality of life for individuals living with COPD.

## Introduction and background

Chronic obstructive pulmonary disease (COPD) is a complex and multifaceted respiratory condition that poses a significant public health challenge globally. Despite being a preventable and treatable disease, it affects a large number of individuals, with approximately 55.3 million diseased people in India alone [1]. The widespread occurrence highlights both its heterogeneous nature and the variety of factors that contribute to its prevalence. Long-term exposure to irritants such as cigarette smoke, air pollution, and occupational hazards remains the primary culprit [2]. These risk factors, coupled with the aging population and lifestyle choices, contribute to the alarming statistics surrounding COPD.

Management of this disease through the phenotype was introduced in the earlier guidelines given by the Global Initiative for Chronic Obstructive Lung Disease (GOLD) [3]. Later, the identification of biological mechanisms resulting from interactions between genetic predispositions and environmental exposures has been the subject of extensive research over the last few decades. This pursuit has led to the emergence of the treatable traits strategy, wherein patients undergo individual assessments to pinpoint treatable issues, guiding the development of personalized treatment programs [4].

Identifiable treatable traits are specific features of COPD that are easily recognizable and can be effectively treated. These traits serve as keys during treatment. They are particularly important where personalized treatment approaches are crucial [5].

The treatable traits strategy acknowledges that multiple traits may coexist within the same patient, advocating for their comprehensive management [6]. This approach signifies a pivotal shift toward deconstructing traditional disease labels like chronic bronchitis and emphysema, opening avenues for identifying candidate traits for chronic airway diseases.

This paper aims to review the common treatable traits and approaches to the identification and treatment of these traits.

## Review

Total heterogeneous nature of COPD

COPD manifests as a heterogeneous lung condition, comprising chronic bronchitis and emphysema. Dyspnea, cough, expectoration, and expectoration are the common clinical manifestations among COPD patients. Because COPD is so complicated, each person is very different, and different biological and physiological processes can cause different clinical presentations. The presentation of these symptoms varies widely in various combinations [7].

The risk factors contributing to COPD development also exhibit heterogeneity, ranging from genetic predisposition to exposure to premature birth, low birth weight, smoke, dust exposure, pollution, pesticide spray, post-infection sequelae, and allergies. The heterogeneity of COPD is further emphasized by the diverse pathological damage observed among patients, ranging from alveolar destruction to airway damage and, in some cases, a combination of both. This destruction is what leads to chronic bronchitis and emphysema, which are the hallmark phenotypic presentations of COPD [8].

Exacerbations of COPD reveal varied types of airway inflammation. Some patients exhibit neutrophilic inflammation, while others display eosinophilic inflammation, highlighting the condition’s heterogeneity. Research also indicates diverse airflow limitations among patients. Fixed airflow limitation, preserved ratio impaired spirometry, pre-COPD stage positioned within the continuum of COPD [9,10].

The complexity of COPD highlights the necessity for the identification of treatable traits, which can guide personalized approaches to diagnosis, treatment, and management. Recognizing the multifaceted aspects of COPD heterogeneity is crucial for advancing research, enhancing clinical outcomes, and tailoring interventions to meet individual patient needs. Understanding the diverse manifestations of COPD facilitates more effective strategies aimed at improving patient care and quality of life [11,12].

The focus of the GOLD guideline committee moves from phenotypic management to treatable traits. The evolution of COPD management over the past two decades highlights the importance of personalized treatment strategies in altering the disease’s natural course. The 2001 GOLD document put a lot of weight on the severity of airflow limitation when deciding how to classify and treat COPD. This was called the forced expiratory volume at first second (FEV1)-centric approach. However, research prompted a shift toward a more patient-centric paradigm, prioritizing symptoms and exacerbation history over FEV1. In 2016, new treatment algorithms were introduced based on identifiable treatable traits and customized follow-up protocols, marking the transition to a patient-centric model [13]. These developments signify a move toward precision medicine in COPD care, emphasizing tailored interventions to meet each patient’s unique needs.

GOLD-endorsed treatable traits

**Figure 1:**
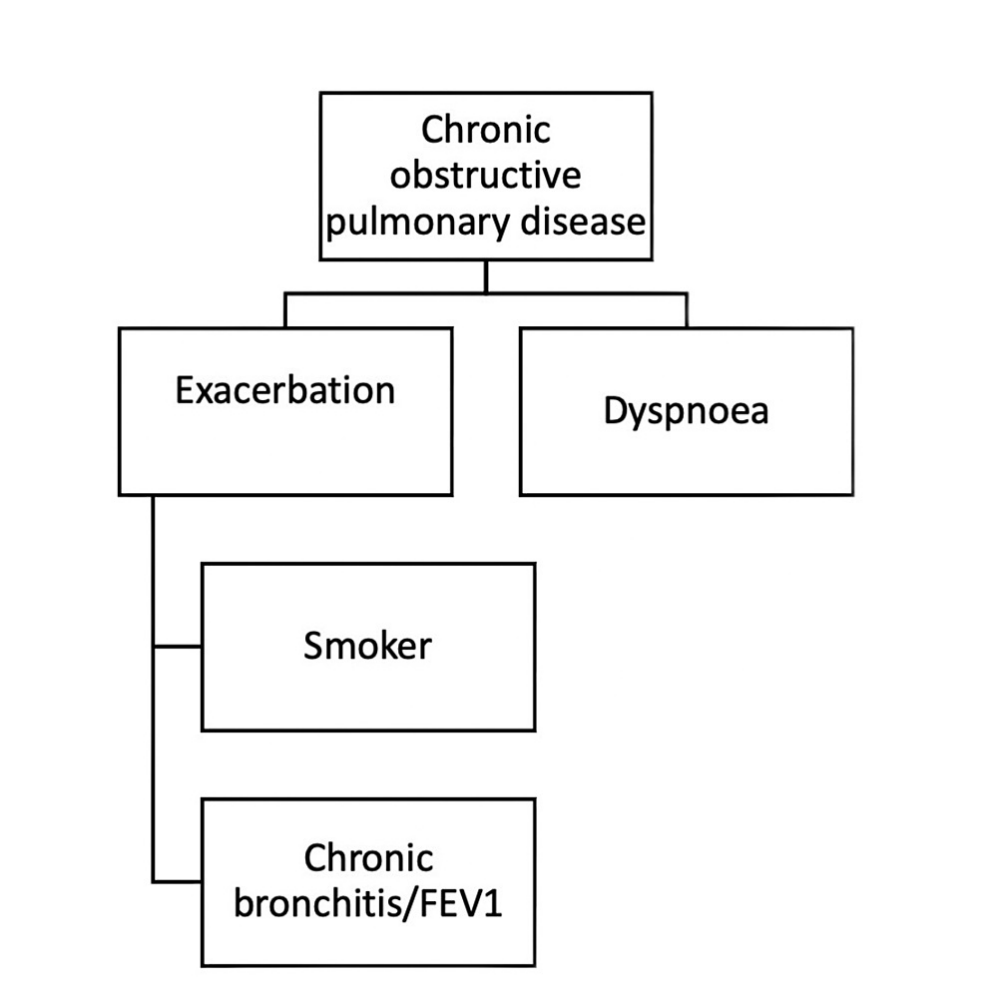
Treatable traits endorsed by the GOLD guideline FEV1, forced expiratory volume at first second; GOLD, Global Initiative for Chronic Obstructive Lung Disease

The concept of identifiable treatable traits is particularly relevant in chronic diseases, where individualized approaches to treatment are increasingly emphasized. GOLD has shifted its focus toward identifiable treatable traits. Among the endorsed basic two identifiable treatable traits are dyspnea and exacerbations (Figure 1).

Dyspnea

Dyspnea, or shortness of breath, is a hallmark symptom of COPD and a key treatable trait that is targeted in COPD management. This significantly impacts the daily lives of COPD patients. The COPD assessment test (CAT) Score and modified Medical Research Council (mMRC) Score serve as valuable biomarkers for assessing dyspnea severity and guiding treatment decisions. Dyspnea in COPD is classified into low and high symptoms based on these scores [7]. The treatment aims to alleviate breathlessness, enhance lung function, and improve exercise capacity, ultimately enhancing patients’ quality of life.

Exacerbations

Exacerbations characterized by sudden symptom worsening pose substantial challenges in COPD management. Exacerbations are another important treatable trait identified by GOLD. Patients with a history of frequent exacerbations are at increased risk. Based on the level of care required, exacerbations are classified into mild, moderate, and severe. Mild exacerbations are those exacerbations that can typically be managed with adjustments to medication or increased use of rescue inhalers at home. Moderate exacerbations may require treatment with corticosteroids and antibiotics, and they may visit an emergency department for evaluation. Severe exacerbations are more severe and may require hospital admission for intensive treatment and monitoring. Exacerbation often involves a significant worsening of symptoms and lung function. Moderate and severe exacerbations have a further risk of exacerbation [14].

Preventing and managing exacerbations is crucial in COPD care to reduce disease progression, hospitalizations, and mortality rates. Strategies for managing exacerbations may include vaccinations, the use of bronchodilators and corticosteroids, and patient education on recognizing early symptoms. Treatment strategies help mitigate exacerbation frequency, alleviate symptoms, and improve lung function, fostering better disease control and patient well-being.

Initial management of COPD

The treatment of COPD is guided by identifiable, treatable traits. Patients with fewer frequent exacerbations are managed with long-acting muscarinic antagonists (LAMA), long-acting beta-agonists (LABA), or their combination. For those experiencing fewer symptoms, short- or long-acting bronchodilators are prescribed, while individuals with higher symptom burdens receive LABA/LAMA combinations [15].

Exacerbations are typically treated with LAMA/LABA alone or in combination with inhaled corticosteroids (ICS), along with azithromycin or roflumilast [16].

Follow-up management of COPD

It is essential to assess adherence to medication, inhaler technique, and the presence of any comorbidities during follow-up visits for COPD patients. Ensuring that patients are adhering to their prescribed medications and using their inhalers correctly can significantly impact disease management and symptom control. It is important to reevaluate and identify any changes in the identifiable treatable traits. This process allows for the adjustment of treatment plans accordingly and ensures that therapy remains aligned with the evolving needs and disease status of the patient.

Patients experiencing dyspnea are typically treated with LABA/LAMA combinations, or adjustments may be made by changing the inhaler device to ensure optimal delivery of medication [17].

For patients experiencing exacerbations, treatment often involves the addition of ICS to their regimen. Additionally, patients with peripheral eosinophilia (>100/ml) may benefit from ICS therapy to help manage inflammation [18].

In the management of COPD, certain other identifiable treatable traits, like smoking history, chronic bronchitis, and low FEV1, play a crucial role in guiding treatment strategies. Among these traits, smoking history and chronic bronchitis represent distinct aspects of the disease process, each requiring specific therapeutic interventions.

Chronic bronchitis is characterized by excessive mucus production and a persistent cough. Patients with chronic bronchitis or FEV1 less than 50% may be treated with roflumilast, a phosphodiesterase-4 inhibitor, to help reduce exacerbation risk and improve lung function by targeting inflammatory pathways associated with mucus production and airway irritation. Roflumilast has demonstrated efficacy in chronic bronchitis by reducing exacerbations and improving lung function [19].

Smoking history is a significant risk factor for COPD and contributes to neutrophilic inflammation within the airways. This inflammatory response is exacerbated by smoking. Often necessitates targeted interventions to reduce exacerbation risk and improve lung function. Treatment may include azithromycin, which can help reduce exacerbations and improve outcomes in this population [20].

Other common treatable traits in COPD

Other common treatable traits in COPD are presented in Table 1.

**Table 1:** Common treatable traits identified in COPD COPD, chronic obstructive pulmonary disease; CSs, corticosteroids; FeNO, fractioned exhaled nitric oxide; FEV1/FVC, forced expiratory volume of first second/forced vital capacity; ICS, inhaled corticosteroids; IgE, serum immunoglobulin E; LABA, long-acting beta-agonist; LAMA, long-acting muscarinic antagonist; Pro-BNP, pro-brain natriuretic peptide

Trait	Biomarker	Intervention	Effect
Smoking [6]	History	Quit smoking/macrolides	Improved symptoms, lung function, and exercise capacity
Dyspnea [6]	COPD assessment test or modified medical research score	LABA/LAMA	Improved symptoms, lung function, and exercise capacity
Exacerbation [6]	History of exacerbation	ICS/LABA/LAMA	Reduced exacerbation, improved symptoms, lung function, and exercise capacity
Airflow limitation [10]	FEV1/FVC ratio <0.7	β_2_-Agonists, antimuscarinic agents, and theophylline	Improved symptoms, lung function, and exercise capacity
Eosinophilic airway inflammation [10]	Serum eosinophil count, IgE, and FeNO	ICSs; oral CSs; anti–IL-5, anti–IL-4, and anti-IL-13; anti-TSLP	Reduced exacerbations, improvement in symptoms, and lung function
Neutrophilic airway inflammation [10]	Induced sputum neutrophil count and CRP	Macrolides and CXCR2 antagonists	Reduced exacerbations and reduced rate of decrease in lung function reduced cough and sputum
Cough reflex hypersensitivity [10]	24-hour cough counts and Leicester Cough Questionnaire	Gabapentin	Improved cough
Mucus overproduction [10]	Sputum production	Carbocisteine	Improved sputum and reduced exacerbations
Bronchiectasis [4]	High-resolution CT	Drainage, macrolides, inhaled antibiotics, vaccination, and surgery	Reduced cough, symptoms, increased exercise capacity, and exacerbations
Pulmonary artery hypertension [4]	2D echocardiography/Pro BNP	Diuretics	Reduced cough, symptoms, and increased exercise capacity
Emphysema [4]	CT scan	Bronchodilator, coils, one-way valve, and lung volume reduction surgery	Improved symptoms, lung function, and exercise capacity

Fixed Airflow Limitation

Airflow limitation is the hallmark of COPD and contributes to impaired lung function and exercise tolerance. The FEV1/FVC ratio <0.7 serves as a vital biomarker for airflow limitation. Interventions, including β2-agonists and antimuscarinic agents, aim to optimize bronchodilation, enhance airflow, improve overall respiratory function, and enhance the ability to perform daily activities with ease [21,22].

Eosinophilic Airway Inflammation

Eosinophilic airway inflammation is characterized by elevated eosinophil counts. Biomarkers such as serum eosinophil count, immunoglobulin E, and fractioned exhaled nitric oxide aid in identifying eosinophilic inflammation. Targeted treatments, including ICS, oral corticosteroids, and biologics targeting IL-5, IL-4, and IL-13, aim to reduce exacerbation risk and improve symptom control, although responses may vary among individuals [23].

Neutrophilic Airway Inflammation

Neutrophilic airway inflammation is a key feature of COPD. It contributes to disease progression and exacerbations. Biomarkers like induced sputum neutrophil count and CRP help identify neutrophilic inflammation. Therapeutic interventions such as macrolides and CXCR2 antagonists target neutrophilic pathways, reducing exacerbation rates, slowing lung function decline, and alleviating cough and sputum production [24].

Cough Reflex Hypersensitivity

Cough reflex hypersensitivity is prevalent in COPD and significantly impacts the quality of life of COPD patients. Biomarkers including 24-hour cough counts and the Leicester Cough Questionnaire quantify cough severity. Gabapentin is an effective treatment, and this desensitizes cough receptors, leading to improved cough control and reduced cough frequency and severity [25].

Mucus Overproduction

Mucus overproduction is also a common trait in COPD. It contributes to airway obstruction and exacerbations. Sputum production serves as a key biomarker for mucus overproduction. Treatment with carbocysteine helps reduce mucus viscosity, promote clearance, and reduce exacerbation frequency, thus improving respiratory symptoms and overall disease control [26].

Emphysema

Emphysema is characterized by the destruction of the lung’s alveoli, leading to impaired gas exchange and respiratory function. The presence and severity of emphysema contribute significantly to disease prognosis and symptomatology. Radiographic imaging, such as CT scans, plays a pivotal role in diagnosing and assessing the extent of emphysema. Treatment strategies aim to alleviate symptoms and slow disease progression. Inhaled bronchodilators, supplemental oxygen therapy, pulmonary rehabilitation, and surgical interventions like lung volume reduction surgery, coils, one-way valves, or lung transplantation may be considered in severe cases [27].

Bronchiectasis

Bronchiectasis involves the abnormal widening and scarring of the airways, leading to recurrent infections and mucus buildup. The presence of bronchiectasis exacerbates respiratory symptoms and complicates disease management. High-resolution CT scans are instrumental in diagnosing and assessing the extent of bronchiectasis. Treatment strategies aim to reduce airway inflammation, control infections, and improve mucus clearance. Antibiotics, bronchodilators, mucolytics, and airway clearance techniques form the cornerstone of bronchiectasis management. Pulmonary rehabilitation and vaccination against respiratory pathogens play roles in preventing exacerbations and improving overall respiratory health [28,29].

Smoking

Smoking history is another prevalent trait among COPD patients. It significantly influences disease severity and progression. The smoking history serves as a crucial biomarker indicating heightened risk and exacerbation potential in COPD. Individuals with a significant smoking history face accelerated lung function decline and increased susceptibility to respiratory infections. Targeted interventions, including azithromycin and smoking cessation programs, address the specific needs of smokers with COPD, aiming to mitigate exacerbation risk and improve long-term outcomes [30,31].

Implementing treatable traits-based COPD management offers several advantages. It allows for tailored treatment plans, addressing specific issues in individual patients, leading to more personalized care and improved outcomes. Target interventions can be applied more effectively by identifying treatable traits such as exacerbation frequency, airflow limitation, or airway inflammation. Regular assessment of treatable traits enables monitoring disease progression and treatment effectiveness more closely, allowing for timely adjustments to the management plan. By considering multiple treatable traits simultaneously, we can adopt a holistic approach to COPD management, addressing various aspects of the disease and its impact on the patient’s health and quality of life [6,13].

Implementing treatable traits-based COPD management offers several drawbacks too. This approach requires comprehensive assessment and monitoring of various clinical parameters, which may be challenging and time-consuming and may require additional resources, including specialized testing, monitoring equipment, and healthcare personnel, which may not be readily available in many healthcare settings. There is currently no standardized approach to identifying and managing treatable traits in COPD, leading to variability in clinical practice and potential inconsistencies in patient care. While the concept of treatable traits in COPD management shows promise, the evidence supporting its efficacy and long-term benefits is still evolving, and further research is needed to establish its effectiveness compared to traditional approaches [32].

## Conclusions

The management of COPD necessitates a comprehensive understanding of its heterogeneous nature and the diverse array of identifiable treatable traits that influence its course. The shift toward identifying and addressing treatable traits marks a significant advancement in COPD management, emphasizing a holistic approach that tailors treatment to individual patient needs. Recognizing and addressing these traits during initial encounters and follow-up visits can effectively optimize symptom control, reduce exacerbation risk, and improve overall disease management. Treatable traits-based COPD management offers several potential benefits, including personalized treatment and targeted interventions. However, its implementation may also pose challenges related to complexity, resource allocation, standardization, and the need for additional evidence.
